# Thermal Degradation Behavior of Thiol-ene Composites Loaded with a Novel Silicone Flame Retardant

**DOI:** 10.3390/polym14204335

**Published:** 2022-10-14

**Authors:** Haonan Chen, Sheng Zhu, Rongfan Zhou, Xintong Wu, Wangyang Zhang, Xiaoshuai Han, Jiangbo Wang

**Affiliations:** 1 School of Materials and Chemical Engineering, Ningbo University of Technology, Ningbo 315211, China; 2 Jiangsu Co-Innovation Center of Efficient Processing and Utilization of Forest Resources, International Innovation Center for Forest Chemicals and Materials, College of Materials Science and Engineering, Nanjing Forestry University, Nanjing 210037, China; 3 Zhejiang Institute of Tianjin University, Ningbo 315201, China

**Keywords:** flame retardancy, thiol-ene, PMDA, thermal degradation

## Abstract

A novel silicone flame retardant PMDA was synthesized and blended with a commercial thiol–ene (TE) to obtain a flame-retardant TE (FRTE) composite. The cone calorimeter measurement showed the incorporation of PMDA improved the flame retardancy of the TE composite at concentrations of 5 wt%. The thermal stability and degradation mechanism of FRTE in nitrogen was studied by thermogravimetric analysis. The degradation behaviour of TE containing a PMDA flame retardant was found to be changed. The kinetics of thermal degradation was evaluated by Kissinger method and Flynn–Wall–Ozawa method. The results showed that the activation energies of the FRTE degradation were higher than those of neat TE. However, the degradation mechanism of the TE matrix was not changed by the incorporation of flame-retardant PMDA. In this study, the flame-retardant mechanism of PMDA flame-retardant TE polymer was explained by using two kinetic analysis methods.

## 1. Introduction

With the continuous progress of research work, various polymerization methods and composite preparation technologies have been developed rapidly [1,2,3]. Photopolymerization is a rapid, inexpensive and simple technique for producing cross-linked materials with thicknesses ranging from a few microns to several millimeters. It is widely used in several applications such as coatings, adhesives, paints, inks, microelectronics, optical materials and dental resins. Because of its many advantages over other polymerization processes including low energy consumption, solvent-free resin compositions, high curing speed and ambient temperature processing, it is considered as a “green” technology [4,5,6].

As a new kind of photopolymerization system, photopolymerized thiol–ene (TE) networks have attracted much interest in academia as well as industries due to unique advantages, such as narrow glass transitions, low shrinkage, and 90% functional group conversion. Thiol–ene photopolymerization is a free radical process rather than a typical chain growth reaction. This photopolymerization is very low under the influence of an oxygen environment and has excellent polymerization stability. However, the flammability of thiol–ene is a serious limitation in areas requiring high flame retardancy [7,8,9,10,11].

In recent years, some different approaches have been reported, and the introduction of boron- and phosphorus-containing compounds into thiol–ene has been shown to be an effective way of improving its flame retardancy [12,13]. Silicon-containing compounds are demonstrated to be one of the choices for use as flame retardants because of their excellent properties, such as high resistance to thermal oxidation, imparting nonflammability, low glass transition temperature, environmental friendliness and low surface energy to the resin [14,15,16,17]. Based on this concept, silicon-containing compounds are expected to be one of the choices for enhancing the flame retardancy of the thiol–ene systems. The methyl group in the structure of silicon-containing compounds can improve the migration performance of a flame retardant during combustion, while the phenyl group can increase the charring performance, so as to achieve efficient flame retardancy.

The flame retardancy of polymer is closely related to its thermal degradation process, which is a chain-breaking process caused by high temperatures or long-time action in the absence of oxygen. In general, thermal degradation is related to the chemical bond energy of polymers. The higher the chemical bond energy, the less likely it is to be degraded. GC-MS, GC-FTIR, etc., can be used for thermal degradation studies [18,19]. In the present work, a novel silicone flame-retardant PMDA was synthesized and introduced into the thiol–ene. PMDA contains methyl, phenyl and amino groups in the structure, forming a body network structure, so that it has excellent flame retardancy and char formation performance in combustion. Here, the flame retardancy and thermal degradation behavior of a thiol–ene/PMDA composite were investigated by cone calorimeter and TGA measurements, respectively.

## 2. Materials and Methods

### 2.1. Materials

Dimethyldimethoxysilane (DMDS) and phenyltrimethoxysilane (PTMS) of reagent grade were supplied from Gelest Chemical Reagent Co., Ltd. (Morrisville, PA, USA). Methyltrimethoxysilane (MTMS), tetramethylammonium hydroxide (TMAOH) and (3-aminopropyl)trimethoxysilane (APS) were all provided by Alfa Aesar Chemical Reagent Co. Ltd. (Tewksbury, MA, USA). Trimethylolpropane tris(3-mercaptopropionate) (3T) was obtained from Bruno Bock Chemische Fabrik Gmblt & Co. (Marschacht, Germany) and used as received. Ethyl alcohol (EtOH), 2,2-dimethoxy-2-phenylacetophenone (DMPA) and pentaerythritol allyl ether (TAE) were purchased from Sigma-Aldrich Reagent Co. Ltd. (St. Louis, MO, USA).

### 2.2. Synthesis of Silicone (PMDA)

**Figure 1:**
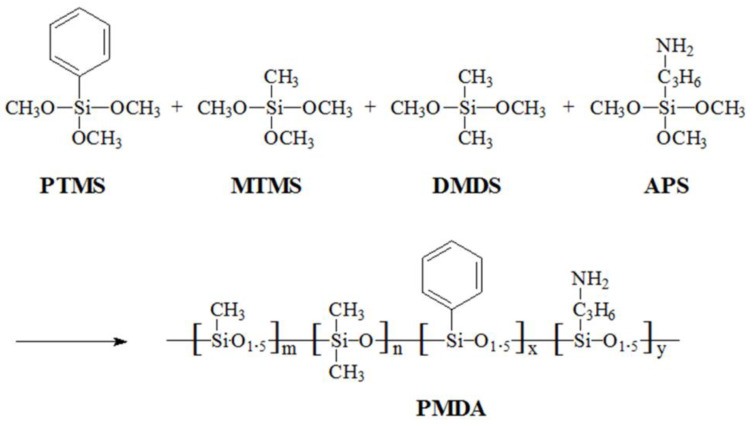
Synthesis of PMDA.

The silicone sample was prepared by hydrolysis and condensation method, which was composed of 60 mol% phenylsiloxane, 35 mol% methylsiloxane and 5 mol% aminosiloxane. The ratio of organic group to silicon atom (R/Si) was 1.2, indicating the degree of branching of polysiloxane structure. The structure of silicone is shown in Figure 1.

In a 250 mL flask, distilled water (25 mL), EtOH (75 mL) and TMAOH (1 mL) were added and stirred. Then, a molar ratio (0.69:0.06:0.20:0.05) of PTMS, MTMS, DMDS and APS mixture was added to the solution, and the total weight percentage was maintained at 10 wt%. The solution was stirred for 8 h and left overnight. The precipitated condensate was collected by decanting the clearest supernatant, washed by vacuum filtration with distilled water/EtOH (1/3 by volume), and then washed again in pure EtOH. The powder (PMDA) was dried and rinsed thoroughly under vacuum for 20 h at room temperature.

### 2.3. Preparation of Thiol-ene Composites

The preparation method of TE/PMDA (FRTE) composite was as follows: The photoinitiator (DMPA, 1 wt%) was dissolved in mercaptan (3 T), and ultrasonically treated for 30 min. Then, TAE (1:1, equal to thiol) and PMDA (5 wt%) were added into the mixed solution, and stirred with a glass rod for 1 min. After further mixing and the removal of bubbles (30 min) ultrasonically, a wire drawing rod was used to pull the uniform mixture onto the glass substrate. The film was cured 10 times under a fused UV-curing line system, under D bulb (400 W/cm^2^, belt speed 3 m/min, irradiance 3.1 W/cm^2^). In order to facilitate comparison, neat TE was also prepared under the same processing conditions.

### 2.4. Characterization and Measurement

Cone calorimeter measurements were performed on an FTT cone calorimeter (Fire Testing Technology Ltd., East Grinstead, West Sussex, UK) with heat flux of 50 kW/m^2^ according to ASTM E1354. The size of each specimen was 100 × 100 × 3 mm^3^. Thermogravimetric analysis (TGA) was performed on a TA instrument Q5000 thermogravimetric analyzer (TA instrument company, New Castle, DE, USA). The sample (approximately 10 mg) was heated in a nitrogen atmosphere from 50 °C to 600 °C at a set heating rate.

## 3. Results and Discussion

### 3.1. Flame Retardancy

**Figure 2:**
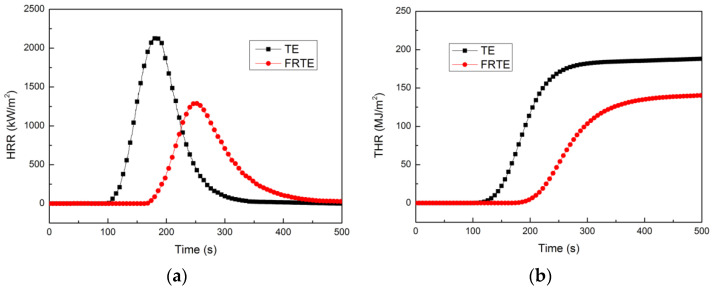
The heat release rate (**a**) and total heat release (**b**) curves for TE composites.

The cone calorimetry uses an oxygen consumption calorimeter to measure the combustion rate and amount of heating. Because the test process is very close to the actual fire situation, it is one of the most effective experimental methods to study the combustion characteristics of polymer materials. Figure 2 illustrates the plots of heat release rate (HRR) and total heat release (THR) versus the temperature of the TE composites. The neat TE exhibited a higher peak of heat release rate (PHRR) of 2152.4 kW/m^2^ and a THR of 188.0 MJ/m^2^. For the FRTE composite, the PHRR was significantly suppressed by 39.4%, from 2152.4 kW/m^2^ to 1304.9 kW/m^2^. Further reduction of THR from 188.0 MJ/m^2^ to 140.3 MJ/m^2^ was observed for FRTE, which was reduced by 25.4%. The decrease of HRR and THR indicated that the incorporation of PMDA into polymer composites could restrict fire development [20,21].

### 3.2. Thermal Stability

**Figure 3:**
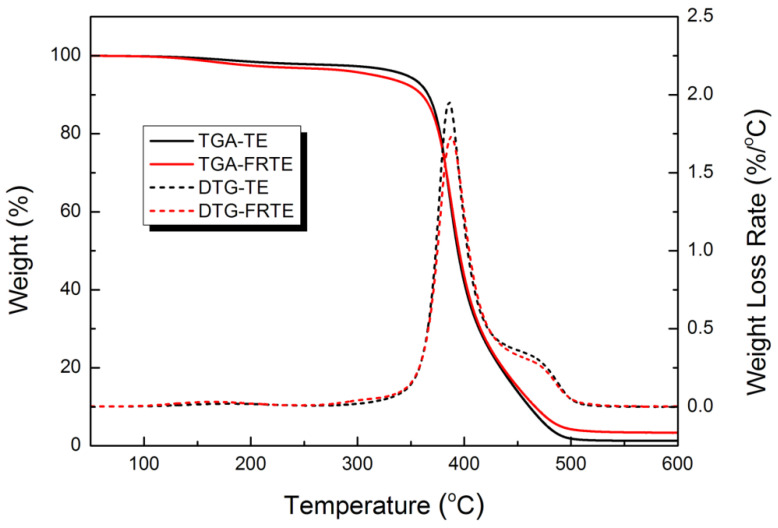
Thermal stability of TE composites.

The fire resistance of materials is closely related to its thermal stability. Currently, TGA is one of the most widely used techniques to rapidly assess the thermal stability of various polymer composites. The thermal properties of TE and FRTE were studied by TGA at heating rates of 10 °C/min under N_2_ atmosphere, as shown in Figure 3 and Table 1. It can be seen that the onset degradation temperature (*T*_5wt%_) of neat TE occurred at about 345.7 °C. With the incorporation of 5 wt% PMDA, the *T*_5wt%_ of FRTE dropped to 316.1 °C, this was mainly due to the condensation reaction of residual silanols and methoxy groups on PMDA. However, the temperature of the peak rate (*T_max_*) for FRTE was slightly higher than that of neat TE, and the main degradation of TE composites was shifted to a higher temperature by the introduction of PMDA.

Moreover, a significant increase in charring was observed in the FRTE, which increased by 2% compared with the neat TE. Therefore, it was apparent from Figure 3 that the PMDA offered significant advantages in the char formation of TE composite.

### 3.3. Thermal Degradation Kinetics

The thermal stability of polymer is related to its initial temperature and degradation rate. Therefore, the kinetic analysis method can be used to calculate the kinetic parameters in the degradation process of the system, which helps to study the thermal stability of the materials. The Kissinger method and Flynn–Wall–Ozawa method are derived from the basic kinetic equation of a multiphase chemical reaction. When obtaining the relevant kinetic parameters, they do not need to determine the reaction order and conversion function, which is very convenient. Therefore, this study will use these two methods to calculate the kinetic parameters of composite materials.

**Figure 4:**
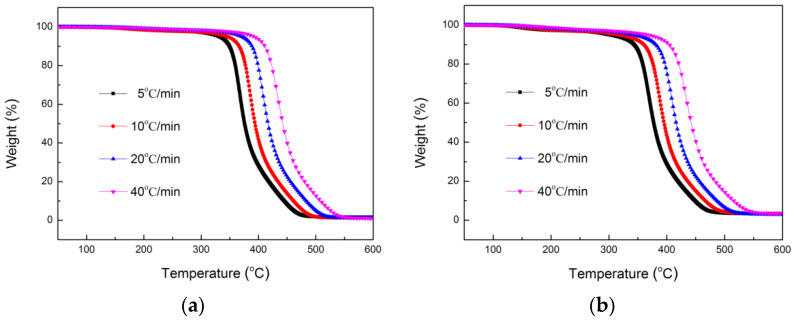
TGA curves of TE (**a**) and FRTE (**b**) composites.

**Figure 5:**
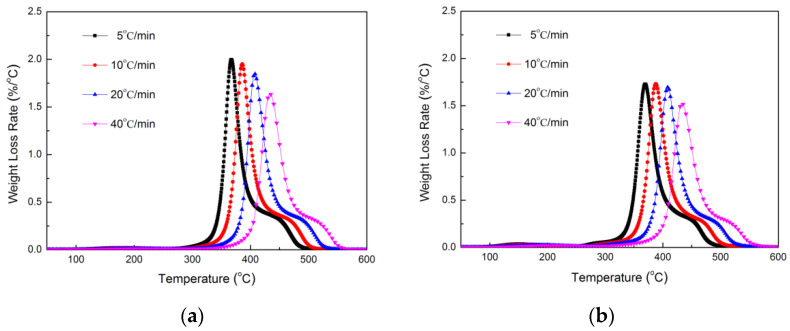
DTG curves of TE (**a**) and FRTE (**b**) composites.

TGA and DTG curves of TE composites in a nitrogen atmosphere were measured at heating rates of 5, 10, 20 and 40 °C/min. As the heating rate increased (Figure 4 and Figure 5), the thermograms shifted to the higher temperature region. At a low heating rate, it was easy to reach equilibrium at any point with the increase of temperature. When the heating rate was too fast, due to slow diffusion of heat, the equilibrium was slow, the curve shifted to the high temperature region, and therefore the degradation point (or degradation temperature) was high.

Kinetic analysis of thermal degradation is one of the potential methods to solve this problem. The kinetic parameters of the whole degradation process were calculated by the Kissinger method. The calculation equation of the Kissinger method is as follows [22]:

(1) $$\ln(\frac{\beta }{T_{\max}^{2}})=\ln(\frac{AR}{E})-\frac{E}{RT_{\max}}$$

where *β* is the heating rate, *A* is the pre-exponential factor, *R* is the universal gas constant and *E* is the apparent activation energy of the kinetic process.

**Figure 6:**
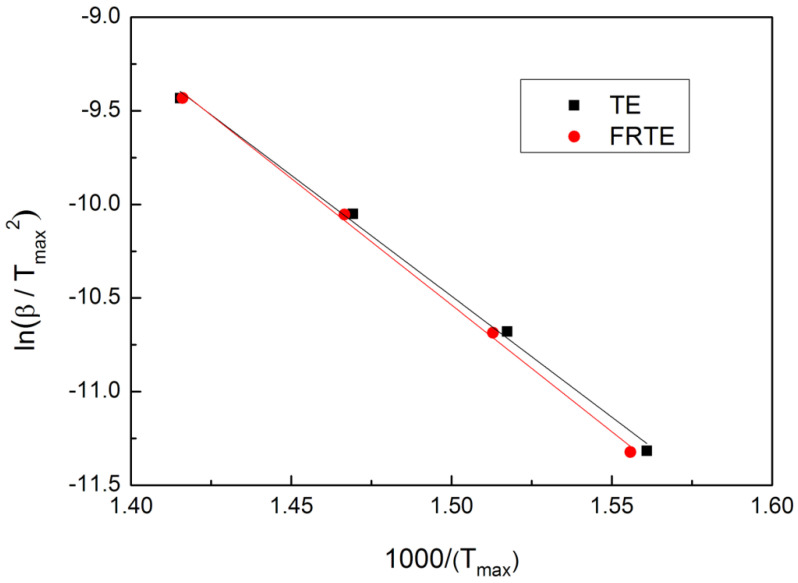
$\ln(\frac{\beta }{T_{\max}^{2}})$ vs. $\frac{1}{T_{\max}}$ curves of TE and FRTE.

The fitting lines of $\ln(\frac{\beta }{T_{\max}^{2}})$ verse $\frac{1}{T_{\max}}$ for the TE composites at various heating rates and the corresponding kinetic parameters calculated by the Kissinger method are shown in Figure 6 and Table 2.

As shown in Table 2, the *E* values of the neat TE and FRTE composites were about 107.4 and 112.5 kJ/mol, respectively. The preexponential factors, *A*, were about 11.4 × 10^10^ and 12.4 × 10^10^/min. It could be obtained from the increase in the apparent activation energy of the TE degradation that PMDA could improve the thermal degradation of TE composite but did not change the degradation mechanism of TE matrix [23].

In order to calculate the degradation kinetics in more detail, the TGA data were also calculated using the Flynn–Wall–Ozawa method. The Flynn–Wall–Ozawa method is not only a typical model-free method, but also a relatively simple and convenient method. The activation energy of the system can be quickly calculated and determined only by obtaining the data of weight loss and temperature of TGA curves at different heating rates.

The equation of Flynn–Wall–Ozawa method is as follows [24,25]:

(2) $$\mathrm{lg}(\beta )=\mathrm{lg}AE/g(a)R-2.315-0.457\frac{E}{RT}$$

**Figure 7:**
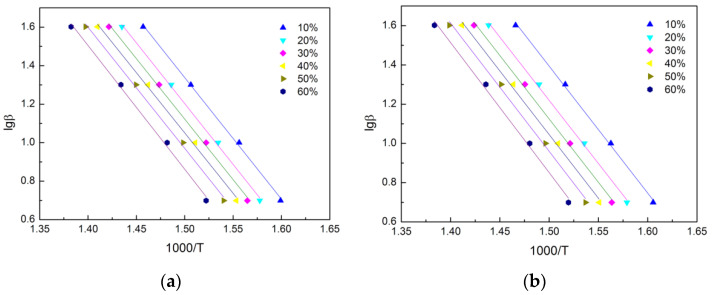
The plots of lg(β) vs. 1000/*T* of TE (**a**) and FRTE (**b**).

According to Equation (2), the activation energies can be calculated by linear fitting of lg(β) verse 1/T plot at different conversion degree. It can be seen from Figure 7 that the fitting lines of the TE and FRTE composites were almost parallel, indicating their unique degradation mechanism. That was, PMDA did not affect the degradation mechanism of TE, which was similar to the results from the Kissinger method.

The apparent activation energy values of neat TE and FRTE composites were obtained by the Flynn–Wall–Ozawa method in the conversion range of 10–60%. The values of conversion 10%, 20%, 30%, 40%, 50% and 60% were used, and the activation energy values corresponding to the different conversion are listed in Table 3. It was found that the *E* of neat TE was lower than that of FRTE composites in the conversion range of 10–60%. The average activation of neat TE and FRTE were 114.9 kJ/mol and 118.2 kJ/mol, respectively.

It can be obtained from the increase in the apparent activation energy of the TE degradation that the incorporation of flame-retardant PMDA could improve the mainly thermal stability of neat TE but did not change the degradation mechanism of TE. Therefore, the increase of char residue amounts of FRTE was important, which were beneficial to the formation of a protective layer in combustion.

## 4. Conclusions

In summary, a novel silicone flame-retardant PMDA, containing methyl, phenyl and amino groups in the structure, was successfully synthesized. Subsequently, it was incorporated into a commercial thiol–ene (TE) to obtain a flame-retardant TE (FRTE) composite. The cone calorimeter measurement showed that the incorporation of PMDA improved the flame retardancy of the TE composite at concentrations of 5 wt%. Compared with the neat TE, the PHRR and THR of FRTE were significantly reduced by 39.4% and 25.4%, respectively. The thermal stability and degradation mechanism of FRTE in a nitrogen atmosphere were studied by thermogravimetric analysis. A significant increase in charring was observed in the FRTE, which increased by 2% compared with the neat TE. Moreover, the kinetics of thermal degradation were evaluated by Kissinger method and Flynn–Wall–Ozawa method. The results showed that the apparent activation energies of the FRTE degradation were higher than those of neat TE. However, the degradation mechanism of the TE matrix did not change by the incorporation of a flame-retardant PMDA.

## Figures and Tables

**Table 1:** TGA data of TE composites.

Sample	Temperature (°C)		Peak Rate (wt%/°C)	Residue Char (wt%)
	*T*_5wt%_	*T_max_*		
TE	345.7	385.9	1.95	1.33
FRTE	316.1	387.8	1.73	3.37

**Table 2:** Kinetic data for TE and FRTE degradation by the Kissinger method.

	Temperature (°C)				*E* (kJ/mol)	ln*A* (1/min)
	5 °C/min	10 °C/min	20 °C/min	40 °C/min		
TE	367.5	385.9	407.5	433.4	107.4	11.4
FRTE	369.6	387.8	408.7	433.0	112.5	12.4

**Table 3:** Activation energies of TE and FRTE degradation by Flynn–Wall–Ozawa method.

a	*E* (kJ/mol)	
	TE	FRTE
0.10	114.8	117.6
0.20	114.8	117.1
0.30	114.3	117.5
0.40	113.9	118.0
0.50	114.5	118.8
0.60	116.9	120.5
Average values	114.9	118.2

## Data Availability

The data used to support the findings of this study are available from the corresponding author upon request.
